# Osthole exhibits an antitumor effect in retinoblastoma through inhibiting the PI3K/AKT/mTOR pathway via regulating the hsa_circ_0007534/miR-214-3p axis

**DOI:** 10.1080/13880209.2022.2032206

**Published:** 2022-02-17

**Authors:** Xiufang Lv, Haojiang Yang, Hui Zhong, Li He, Li Wang

**Affiliations:** a Department of Ophthalmology, Shenzhen Children’s Hospital, Shenzhen, Guangdong, China; b Department of Ophthalmology, The First Affiliated Hospital of Shenzhen University, Shenzhen Second People's Hospital, Shenzhen, Guangdong, China

**Keywords:** Osthole, hsa_circ_0007534, miR-214-3p, retinoblastoma, PI3K/AKT/mTOR pathway

## Abstract

**Context:**

Osthole shows antitumor effects in various tumours. Studies describing the effect of osthole on retinoblastoma (RB) are rare.

**Objective:**

This study investigates the antitumor activity of osthole on RB.

**Materials and methods:**

RB cells were treated with different concentrations of osthole and then subjected to cell viability, colony formation, apoptosis, and western blot assays. The expression of hsa_circ_0007534 in RB tissues was determined by qRT-PCR. Hsa_circ_0007534 overexpression plasmid (oe-circ_0007534), miR-214-3p mimics and negative controls were transfected into RB cells to investigate cell viability. Athymic nude mice were injected with Y-79 cells to establish subcutaneous RB models. These mice were treated with osthole (0.5 mmol/kg) or corn oil for 36 days. Tumour tissues were collected for further analysis.

**Results:**

Osthole inhibited cell viability of RB cells with an IC_50_ of 200 μM for 24 h treatment and 120 μM for 48 h treatment, respectively. Hsa_circ_0007534 was increased significantly in RB tissues as compared to the matched nontumor tissues (*p* < 0.001). Oe-circ_0007534 counteracted the inhibitory effect of osthole on cell viability and colony numbers of Y-79 cells (*p* < 0.01). *In vivo* experiments indicated osthole significantly decreased the expression of hsa_circ_0007534 (*p* < 0.01) and increased the level of miR-214-3p *in vivo.* Furthermore, as compared to the control, osthole decreased the ratios of p-PI3K/PI3K, p-AKT/AKT and p-mTOR/mTOR (*p* < 0.01). However, hsa_circ_0007534 overexpression reversed the effect of osthole on the PI3K/AKT/mTOR pathway.

**Discussion and conclusions:**

Osthole exhibited an antitumour effect in RB, providing a scientific basis for further research and clinical applications of osthole in RB treatment.

## Introduction

Retinoblastoma (RB) is the most common primary intraocular neoplasm in children (Su et al. 2018). It constitutes 3% of all childhood cancers (Pekacka 2020). For the treatment of RB, a major challenge is the metastatic and secondary tumours that often occur later in life (Silva et al. 2010). Thus, it is imperative to discover and develop novel therapeutic agents with better efficiency and fewer adverse effects.

**Figure 1.**
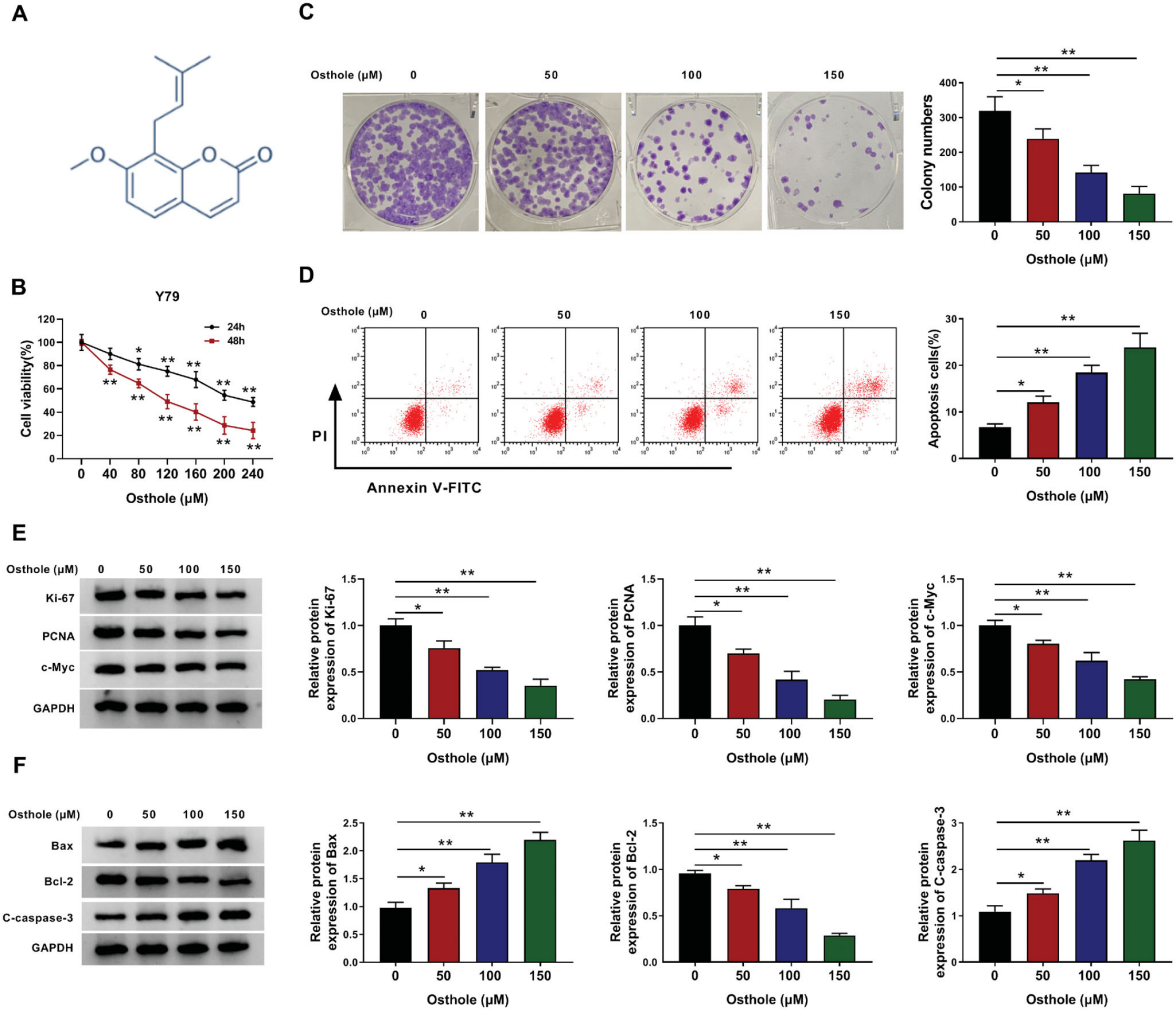
Osthole inhibited cell viability, proliferation, colony formation and induced apoptosis in Y-79 cells. (A) The structure of osthole. (B) Y-79 cells were treated with or without osthole for 24 or 48 h and then subjected to the cell viability CCK8 assay. (C) The colony forming ability of Y-79 cells after osthole treatment was measured by clonogenic assays. (D) The apoptosis rate of Y-79 cells after osthole treatment for 48 h was measured by tumour cell apoptosis assay. (E) The expression of proliferation markers was determined by western blot analysis. (F) The expression of apoptosis-related proteins was determined by western blot analysis. *p < 0.05, **p < 0.01.

Natural products, containing bioactive secondary metabolites, have beneficial effects on human health. Osthole [7-methoxy-8-(3-methyl-2-butenyl) coumarin, Figure 1A] is a natural coumarin first derived from *Cnidium* plant. Osthole frequently presents in the mature fruit of *Cnidium monnieri* Cusson ex Juss (Fructus Cnidii) of the Apiaceae family. Osthole is known to exert anti-inflammatory, antimicrobial, and anti-allergic activities (Matsuda et al. 2002; Shokoohinia et al. 2018). In addition, osthole is also known to exert therapeutic effects against several cancer types including breast (Park et al. 2019), gastric (Xu et al. 2018), gallbladder (Le Zou et al. 2019) and ovarian cancers (Bae et al. 2021). It has been reported that osthole exerts antitumor effects by inhibiting cell proliferation, inducing cell cycle arrest and apoptosis, and inhibiting the epithelial-mesenchymal transition (EMT) process (Wen et al. 2015; Xu et al. 2018; Zhu et al. 2018). The signalling pathways, such as PI3K/AKT and JAK/STAT3, have been reported to be involved in the antitumor process of osthole (Zhu et al. 2018; Le Zou et al. 2019). Although many cancer types were reported to be inhibited by osthole, the effect of osthole on RB still remains unclear.

CircRNA is a class of endogenous non-coding RNA and exists ubiquitously in the cytoplasm of eukaryotic cells (Zhang et al. 2018). Unlike other linear RNAs, circRNAs lack the 3′ and 5′ ends, which protects it from the degradation by RNA exonuclease, so they can be more stably presented in tissues and cells (Zhang et al. 2020). CircRNAs perform a wide variety of biological functions in eukaryotic cells primarily through competing endogenous RNAs (ceRNAs) or miRNA sponges (Wang et al. 2020). Growing evidence has indicated that circRNAs play important roles in tumours, involving multiple processes, including proliferation, EMT, apoptosis, and cell cycle regulation (Harper et al. 2019; Li et al. 2020). In RB, overexpression of circRNAs, such as circ_0000034 and circ_0000527, has been observed (Wang et al. 2020; Zhang et al. 2020). These molecules are involved in the development of RB (Wang et al. 2020). The roles and mechanisms of other circRNAs in RB should be clarified as well.

Hsa_circ_0007534 is located at chr17: 61869771–61877977 and is a transcription product of the DEAD-box helicase 42 (DDX42) gene (Qi et al. 2018). Recently, hsa_circ_0007534 was identified as a potential cancer-associated circRNA. Qi et al. (2018) found that hsa_circ_0007534 was a potential lung cancer-associated prognostic/therapeutic target. Ding et al. (2020) demonstrated that hsa_circ_0007534 was up-regulated in colorectal cancer (CRC) and hsa_circ_0007534 knockdown could suppress the growth of CRC cells. Rong et al. (2019) indicated that downregulation of hsa_circ_0007534 could repress the proliferation and invasion of cervical cancer. Thus, hsa_circ_0007534 could increase cancer cell proliferation, migratory, invasion, and inhibit cell apoptosis. Downregulation or knockdown of hsa_circ_0007534 may restrict the proliferation and invasion of cancer cells significantly. However, the link between hsa_circ_0007534 expression and RB has not been determined previously.

In the present study, the antitumor activity of osthole in RB was investigated *in vitro* and *in vivo.* The underlying molecular events of osthole treatment on RB were detected. Furthermore, the biological functions of hsa_circ_0007534 in RB and the regulating effect of osthole on the hsa_circ_0007534 axis were also determined.

## Materials and methods

### Patients and specimens

Forty pairs of RB tumour tissues and matched adjacent non-tumorous tissues were collected from patients who had undergone surgery at the Department of Ophthalmology, Shenzhen Children’s Hospital (Shenzhen, China) between Jan 2016 and Sep 2018. All collected tissue samples were immediately stored in liquid nitrogen. Human samples were obtained with written informed consent from all patients. The study was approved by the Ethics Committee of Shenzhen Children’s Hospital (Shenzhen, China).

### Cell culture and treatment

Y-79 human RB cell line was obtained from the Chinese Academy of Science Cell Bank (Shanghai, China). A human retinal pigment epithelial (RPE) cell line (ARPE-19) was obtained from ATCC (American Type Culture Collection). Y-79 cells were cultured in RPMI-1640 medium (Gibco, Grand Island, NY, USA), accompanied by 10% foetal bovine serum (FBS) and 1% antibiotic–antimycotic solution penicillin-streptomycin (Corning Incorporated, Corning, NY, USA). ARPR-19 cells were grown in Dulbecco's modified Eagle's medium (DMEM; Gibco) supplemented with 10% FBS (Gibco), 1% streptomycin and 1% penicillin. All types of cells were cultured in a humidified atmosphere of 5% CO_2_ at 37 °C.

Osthole powder (purity > 98%) was purchased from Solarbio Science & Technology (Beijing, China). Osthole was dissolved in DMSO and then diluted in medium to the desired final concentration (0, 40, 50, 80, 100, 120, 150, 160, 200, or 240 μM). Y-79 cells were treated with osthole at increasing concentrations at different times. In pathway activation and inhibition tests, Y-79 cells were pre-treated with 740Y-P (30 μM) for 1 h and then incubated with osthole (150 μM) for 48 h before analysis.

### Cell viability assay

The effect of osthole on the viability of Y-79 cells was assessed using the CCK-8 assay according to the manufacturer's instructions (Dojindo Molecular Technologies Inc., Kumamoto, Japan). Briefly, Y-79 cells were seeded at 1 × 10^5^ cells/mL density, in 96-well plates. Then, Y-79 cells were treated with osthole (0, 40, 80, 120, 160, 200, or 240 μM) for 24 or 48 h. After osthole treatment, 10 μL of CCK-8 solution was added, and the cells were placed in an incubator at 37 °C for 1 h. Cell proliferation was determined by measuring the optical density at 450 nm.

### Tumour cell colony formation assay

Cells were seeded into 6-well plates at a density of 1,000/well, grown overnight and then treated with osthole (0, 50, 100, or 150 μM) for 12 days. At the end of the experiments, the cells were fixed with methanol (Sangon Biotech Co., Ltd., Shanghai, China) for 20 min and stained with 0.5% crystal violet (Sangon Biotech). Cell colonies with ≥50 cells were counted using an inverted microscope (Leica Microsystems GmbH, Wetzlar, Germany) in five randomly selected fields.

### Tumour cell apoptosis assay

Cells were seeded at a density of 10^6^ cells/well in 12-well plates. After 48 h treatment with osthole (0, 50, 100, or 150 μM), cells were collected and incubated with fluorescein isothiocyanate conjugated anti-annexin V antibody and propidium iodide (PI) according to the manufacturer’s protocol (KeyGen Biotech, Nanjing, China). Apoptotic cells were separated and quantified by a FACSCalibur Flow Cytometry System (Becton Dickinson, San Jose, USA).

### RNA extraction and quantitative real-time PCR (qRT-PCR)

Total RNAs were extracted from cells and tissues using TRIZOL reagent (Invitrogen). RNA was reverse-transcribed using the cDNA Synthesis Kit (TaKaRa Bio, USA). qRT-PCR was performed with an ABI StepOnePlus™ real-time PCR System (Applied Biosystems, USA) using the SYBR Green mix (Toyobo, Japan). Glyceraldehyde-3-phosphate dehydrogenase (GAPDH) was used as an internal control for normalisation. U6 was used as an internal control for miRNA expression. The relative gene expression was calculated using the 2^-ΔΔCt^ method. The PCR primers were used in this study as follows: hsa_circ_0007534, forward 5′-GTGACGGAAATCCAATTGCACC-3′ and reverse 5′-ATGGAATTGCTGGCGAGTTG-3′; DDX42, forward 5′-TCTCTCGAGGCTGAAGTGGAGGATCAGGCTGC-3′ and reverse 5′-CGCGGATCCCTAACTATCCCATCGGCTTTTC-3′; miR-214-3p 5′-ACAGCAGGCACAGAGACCGGCAGU-3′ and 3′-UGUCGUCCGUGUCUGUGUCCGUCA-5′; GAPDH, forward 5′-GGGAGCCAAAAGGGTCAT-3′ and reverse 5′-GAGTCCTTCCACGATACCAA-3′; U6, forward 5′-TGCGGGTGCTCGCTTCGGCAGC-3′ and reverse 5′-CCAGTGCAGGGTCCGAGGT-3′.

### Western blot analysis

The proteins extracted from tissues and cultured cells were separated through SDS‐PAGE and then transferred onto polyvinylidene fluoride (PVDF) membranes (Millipore, Billerica, MA, USA). After blocked for 1 h for non-specific binding in Tween 20 (TBST) with 5% non-fat milk, blots were incubated with primary antibodies overnight at 4 °C. Antibodies against Ki67 (1:1,000, Cat # ab 92742, Abcam, Cambridge, UK), proliferating cell nuclear antigen (PCNA) [1:1,1000, Cat # 13110, Cell Signalling Technology (CST), Danvers, MA, USA], c-Myc (1:1,1000, Cat # ab10910, Abcam), Bcl-2 (1:1,1000, Cat # 4223, CST), Bcl2-associated X protein (Bax) (1:1,1000, Cat # 5023, CST), Cleaved caspase 3 (1:1,000, Cat # ab13847, Abcam), PI3K (1:1,000, Cat # 4255, CST), p-PI3K (1:1,000, Cat # 17366, CST), AKT (1:1,000, Cat # 9271, CST), p-Akt (1:2,000, Cat # 9611, CST), mTOR (1:1,1000, Cat # 2983, CST), p-mTOR (1:1,1000, Cat # 5536, CST) and GAPDH (1:1,000, Cat # 5174, CST) were incubated. Then, membranes were incubated with antirabbit IgG–HRP-conjugated secondary antibodies (1:5,000, Wuhan Boster Biological Technology, Wuhan, China) for 1 h at room temperature. The bound secondary antibodies on the PVDF membrane were reacted with ECL detection reagents (Beyotime, Shanghai, China) according to the manufacturer's protocol. GAPDH was used as an internal loading control.

### Cell transfection

The has_circ_0007534 overexpression plasmid (oe-circ_0007534) was constructed using pCD5‐ciR vector (Geneseed Biotech Co., Ltd., Guangzhou, China). Meanwhile, a control plasmid (vector) was also constructed. The miR-214-3p mimics and negative controls (miR-NCs) were designed and obtained from GenePharma (Shanghai, China). Small interfering RNA (siRNA) against hsa_circ_0007534 (si-circ_0007534) and scrambled negative control (si-NC) were provided by RiboBio Co., Ltd. (Guangzhou, China). The aforementioned plasmids were transfected into Y-79 cells using Lipofectamine 2000 (Invitrogen) according to the manufacturer's instructions. After transfection for 48 h, transfected cells were treated with different concentrations of osthole for an additional 48 h. Then, cell-viability assay, cell apoptosis assay, cell colony formation assay, qRT-PCR and western blot analysis were performed as the methods described above.

### RNase R digestion

Total RNA isolated from Y-79 cells were incubated with 6 units of RNase R (Epicenter Biotechnologies, Shanghai, China). Total RNA untreated with RNase was used as a control (mock). After total RNA and RNase R incubation for 15 min at 37 °C, the levels of hsa_circ_0007534 and DDX42 mRNA were examined using qRT-PCR.

### Actinomycin D assay

After Y-79 cells (5 × 10^4^ cells/well) were seeded into 24-well plates and incubated overnight, 2 μg/mL actinomycin D (Abcam) was added to block transcription at indicated time points (0, 4, 8, 12 and 24 h). Next, qRT-PCR was conducted to examine the expression of hsa_circ_0007534 and DDX42 mRNA.

### Subcellular fractionation location

RNA was isolated from the nuclear or cytoplasmic fraction using the Nuclear/Cytosol Fractionation Kit (Biovision, San Francisco Bay, CA, USA) and was measured by qRT-PCR. U6 and GAPDH acted as the identifiers for the nuclear or cytoplasmic fractions, respectively. Nuclear and Cytoplasmic Extraction Reagents were purchased from Thermo Fisher Scientific.

### Bioinformatic prediction and dual-luciferase reporter assay

The targets of hsa_circ_0007534 and miR-214-3p were predicted by Starbase3.0 (http://starbase.sysu.edu.cn). The wild type (WT) and mutant (MUT) of circ_0007534 were constructed and inserted into luciferase reporter assay vector pmirGLO (Promega Corp., Madison, WI, USA) as described previously (Meng et al. 2021). The Y-79 cells were co-transfected with 10 nM miR-NC or miR-214-3p and 40 ng circ_0007534 WT or circ_0007534 MUT. After 48 h transfection, cells were harvested and the luciferase activity was detected with the dual-luciferase reporter assay system kit (Promega) using the luminometer (Plate Chameleon V, Hidex, Finland) according to the manufacturer’s instructions.

### RNA pull-down assay

Y-79 cells were transfected with the wild type biotin-labelled miR-214-3p (Bio-miR-214-3p), mutant biotin-labeled miR-214-3p (Bio-miR-214-3p-mut), and non-specific negative control (Bio-miR-NC). After 48 h, cell lysates were incubated with streptavidin magnetic beads (Life Technologies, Mountain View, CA, USA), followed by qRT-PCR to measure the level of has_circ_0007534 in RNA complexes.

### *In vivo* tumour growth assay

Experiments were performed under a project licence granted by the Animal Care and Use Committee of Shenzhen University, in compliance with the Guide for the Care and Use of Laboratory Animals (NIH Publication 86-23, National Academic Press, Washington, DC, USA, 1996). Four-week-old male Nu/nu athymic nude mice obtained from Weitonglihua Biotechnology (Beijing, China) were raised under specific pathogen-free conditions. The subcutaneous RB model was established by injecting 100 μL suspension of Y-79 cells (1 × 10^7^ cells/mL) into the right flank of the athymic nude mouse. One week post-implantation, 12 mice (with visible tumours) were randomly divided into two groups (osthole and control), with 6 mice in each group. Each mouse was treated with either 0.5 mmol/kg of osthole in 0.2 mL corn oil (osthole group) or the same volume of corn oil alone (control group) through intraperitoneal injection once every day for 36 days. During the treatment, tumour size was measured by callipers (length and width) every 3 days. The tumour volumes were calculated with the formula *V* = a × b^2^ × π/6, where a is the larger and b is the perpendicular shorter tumour axis. All animals were sacrificed on day 36 after treatment. Then, tumour weight of each mouse was immediately measured. The tumour tissues were collected for further analysis including qRT-PCR, western blot analysis and immunohistochemistry.

Next, another 30 mice were used to determine the role of osthole on PI3K/AKT/mTOR pathway *in vivo*. Y-79 cells were transfected with oe-circ_0007534, vector, oe-circ_0007534 + miR-NC and oe-circ_0007534 + miR-214-3p for 48 h, respectively. Then, a total of 1 × 10^6^ cells (normal Y-79 cells or transfected cells) were injected into the right flank of the nude mouse. The tumour-bearing mice were randomly assigned to six groups (*n* = 5): control, osthole, osthole + vector, osthole + oe-circ_0007534, osthole + oe-circ_0007534 + miR-NC and osthole + oe-circ_0007534 + miR-214-3p. For 5 osthole-treated groups, each mouse was treated with 0.5 mmol/kg of osthole through intraperitoneal injection once every day. For control group, each mouth was treated with the same volume of corn oil alone. Mice in each group were sacrificed 4 weeks after injection, and the tumour nodules were harvested for western blot analysis.

### Immunohistochemistry

Tumour tissue samples were fixed in 10% formalin, paraffin-embedded and sectioned. Tissue sections with 5 mm thick were dewaxed and incubated with 0.01 M sodium citrate for antigen retrieval. The slides were incubated overnight at 4 °C with rabbit antimouse Ki67 or cleaved caspase 3 primary antibodies (CST). Biotinylated goat anti-rabbit anti-immunoglobulin G (Wuhan Boster Biological Technology) was used as the secondary antibody.

### Statistical analysis

SPSS22.0 software (Chicago, IL, USA) was used for statistical analyses. All results were presented as mean ± standard deviation (SD). Differences between groups were evaluated using a two-tailed Student’s *t*-test or one-way analysis of variance (ANOVA). The two‐way repeated ANOVA was also performed to analyse the time × treatment interaction. *p* < 0.05 were considered to be significant.

## Results

### Osthole inhibited cell viability, proliferation, colony formation and induced apoptosis in Y-79 cells

It was found that osthole reduced the viability of the Y-79 cells (Figure 1B). When Y-79 cells were treated with osthole for 24 h, the IC_50_ value of osthole was 200 μM. When Y-79 cells were treated for 48 h, the IC_50_ value of osthole was 120 μM. The results of the colony formation assay are shown in Figure 1C. As the concentration of osthole increased (0, 50, 100, and 150 μM), the proliferation of Y-79 cells was significantly reduced (Figure 1C). The role of osthole on cell apoptosis was detected by flow cytometry. Our results showed that osthole treatment increased the apoptotic rate of the Y-79 cells (*p* < 0.05, Figure 1D). Furthermore, osthole significantly reduced the expressions of Ki67, PCNA and c-Myc (Figure 1E). For apoptosis-related proteins, the levels of Bax and cleaved caspase 3 were increased after osthole treatment. Conversely, the level of B-cell lymphoma 2 (Bcl-2) was decreased in osthole-treated groups compared to the control (*p* < 0.05, Figure 1F).

### Osthole played a role on Y-79 cell proliferation and apoptosis through inhibiting PI3K/AKT/mTOR pathway

**Figure 2.**
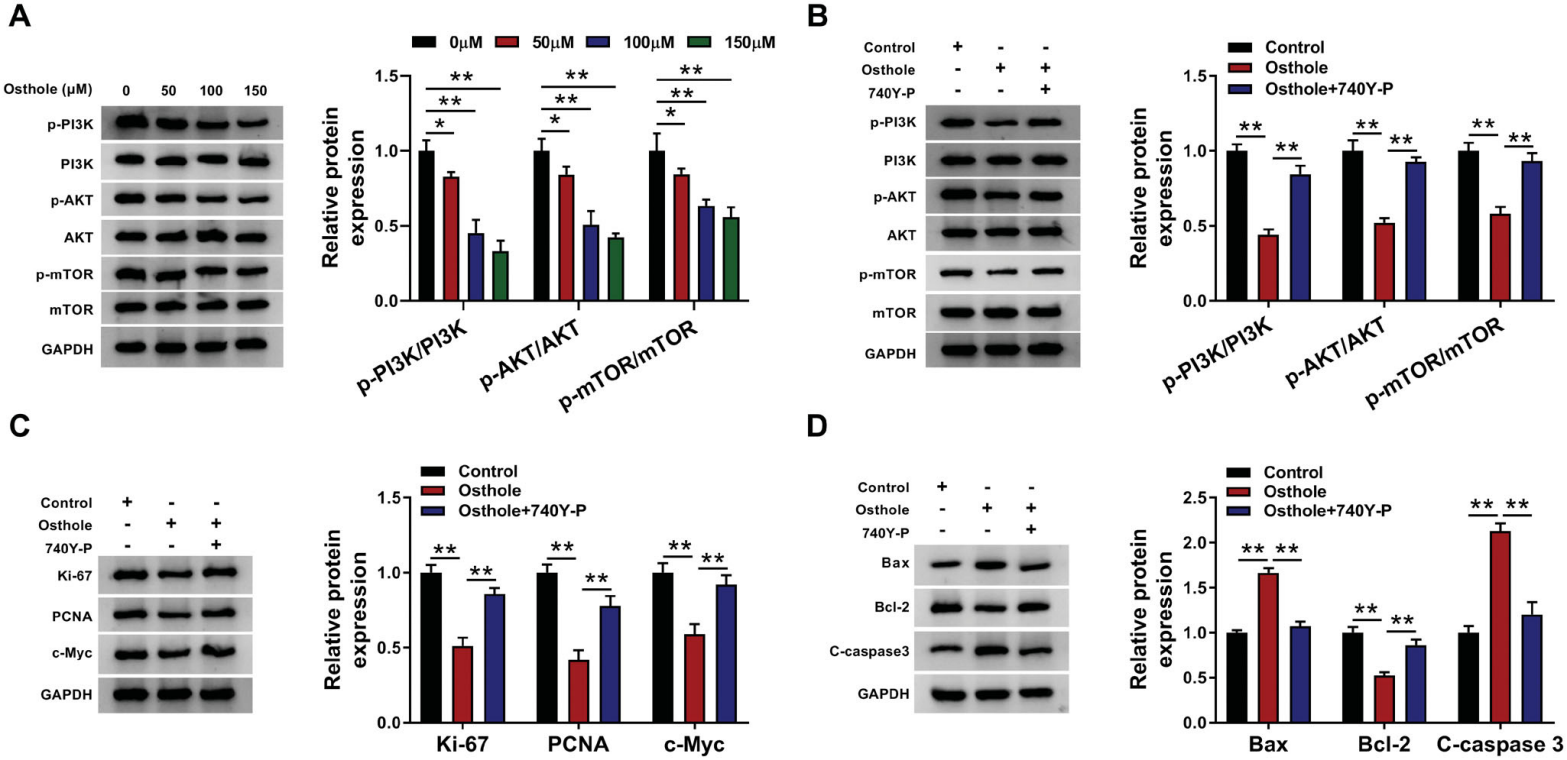
Osthole regulated Y-79 cell proliferation and apoptosis by inhibiting PI3K/AKT/mTOR pathway. (A) Y-79 cells were treated with different concentrations of osthole, and the expression of components in PI3K/AKT/mTOR pathway was determined by western blot analysis. (B) Y-79 cells were treated with osthol or osthol + 740Y-P, and the expression of components in the PI3K/AKT/mTOR pathway was determined by western blot analysis. (C) Y-79 cells were treated with osthol or osthol + 740Y-P, the expression of proliferation markers was determined by western blot analysis. (D) Y-79 cells were treated with osthol or osthol + 740Y-P, the expression of apoptosis-related proteins was determined by western blot analysis. *p < 0.05, **p < 0.01.

To detect the role of osthole on PI3K/AKT/mTOR pathway, Y-79 cells were treated with different concentrations of osthole. After osthole treatment, the p-PI3K/PI3K, p-AKT/AKT, and p-mTOR/mTOR ratios were decreased (Figure 2A). Then, to further investigate the role of osthole on PI3K/AKT/mTOR pathway, cells were treated with osthole, osthole + 740Y-P, and control, respectively. As expected, the ratios of p-PI3K/PI3K, p-AKT/AKT, and p-mTOR/mTOR were lower in the osthole group, with a significant difference compared to the control (*p* < 0.01). And 740Y-P reversed the effect of osthole on PI3K/AKT/mTOR pathway (*p* < 0.01, Figure 2B). These findings demonstrated that osthole could inhibit PI3K/AKT/mTOR signalling activity. Furthermore, the cell proliferation-related proteins (Ki67, PCNA and c-Myc) were higher in osthole + 740Y-P group compared with osthole group (*p* < 0.01, Figure 2C). 740Y-P also reversed the effect of osthole on apoptosis-related proteins (Figure 2D).

### Hsa_circ_0007534 was highly expressed in RB tissues and cells

**Figure 3.**
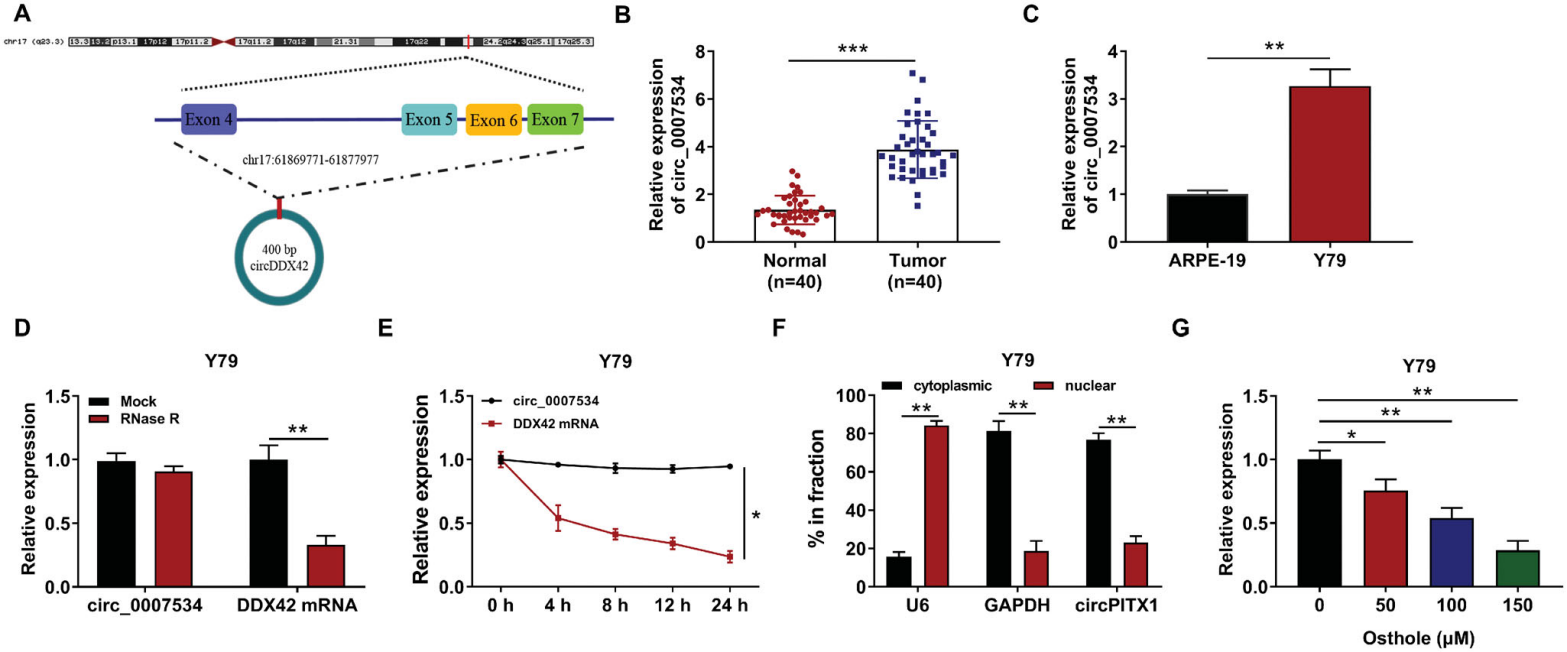
Hsa_circ_0007534 was highly expressed in RB tissues and cells. (A) Hsa_circ_0007534 is derived from exons 4 to 7 of the DDX42 gene. (B) Hsa_circ_0007534 expression in retinoblastoma tissues and normal tissues was detected by qRT-PCR. (C) Hsa_circ_0007534 expression in Y-79 cells and control cells was detected by qRT-PCR. (D) qRT-PCR was performed to analyse the hsa_circ_0007534 and DDX42 level after treatment with RNase R. (E) qRT-PCR was performed to analyse the hsa_circ_0007534 and DDX42 level after treatment with Actinomycin D. (F) Subcellular localisation of hsa_circ_0007534 was determined by qRT-PCR. (G) Y-79 cells were treated with different concentrations of osthole, and the expression of hsa_circ_0007534 was detected by qRT-PCR. *p < 0.05, **p < 0.01, ***p < 0.001.

Hsa_circ_0007534 is derived from exons 4 to 7 of the DDX42 gene, whose spliced mature sequence length is 400 bp (Figure 3A). We found that the expression of hsa_circ_0007534 was increased significantly in RB tumour tissues as compared to the matched nontumor tissues (*p* < 0.001, Figure 3B). Furthermore, compared with normal human retinal pigment epithelial cells (ARPE-19), the expression of hsa_circ_0007534 was much higher in Y-79 cells (*p* < 0.01, Figure 3C). Afterward, the circular characteristics of hsa_circ_0007534 were investigated using RNase R and actinomycin D assays. RNase R assay indicated that hsa_circ_0007534 was resistant to RNase R, while linear DDX42 mRNA was distinctly digested by RNase R (Figure 3D). The results of the actinomycin D assay showed that hsa_circ_0007534 was more stable than linear DDX42 mRNA in Y-79 cells (*p* < 0.05, Figure 3E). The cell fraction assay showed that hsa_circ_0007534 was primarily located in the cytoplasm and also existed in the nucleus (Figure 3F). Collectively, hsa_circ_0007534 was a circular and stable transcript and was upregulated in RB. Then, Y-79 cells were treated with different concentrations of osthole for 48 h, and the expression of hsa_circ_0007534 was detected by qRT-PCR. Our results showed that osthole treatment reduced the expression of hsa_circ_0007534 (Figure 3G).

### Has_circ_0007534 functioned as an efficient miR-214-3p sponge

**Figure 4.**
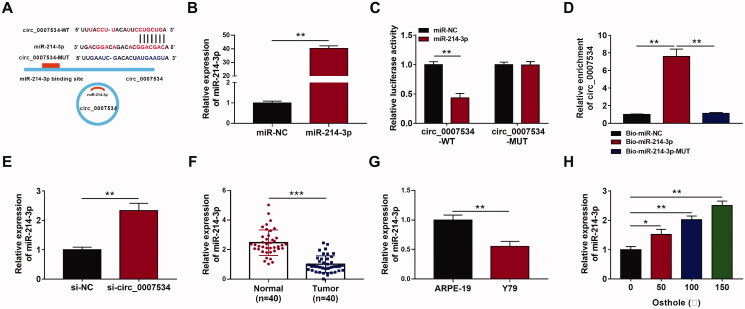
Has_circ_0007534 functioned as an efficient miR-214-3p sponge. (A) A diagram showed the predicted binding site of miR-214-3p in hsa_circ_0007534. (B) Cells were transfected with miR-214-3p mimics or miR-NC, and the expression of miR-214-3p was detected by qRT-PCR. (C) Luciferase reporter assay detected luciferase activity after co-transfecting with a miR-214-3p or miR-NC and circ_0007534-WT or circ_0007534-MUT reporter plasmid. (D) RNA pull-down assay detected the expression of hsa_circ_0007534 in the RNA complexes. (E) Knockdown of hsa_circ_0007534 upregulated miR-214-3p levels. (F) miR-214-3p expression in retinoblastoma tissues and normal tissues was detected by qRT-PCR. (G) miR-214-3p expression in Y-79 cells and control cells was detected by qRT-PCR. (H) Y-79 cells were treated with different concentrations of osthole, and the expression of miR-214-3p was detected by qRT-PCR. *p < 0.05, **p < 0.01, ***p < 0.001.

Starbase 3.0 was utilised to predict the potential pairing bases between has_circ_0007534 and miR-214-3p. And the result showed a putative targeting site of miR-214-3p in the has_circ_0007534 transcript (Figure 4A). The level of miR-214-3p was significantly elevated in Y-79 cells transfected with miR-214-3p mimics compared with miR-NC (*p* < 0.01, Figure 4B). Wt and Mut circ_0007534 were constructed for the dual-luciferase reporter assay. Our results showed that luciferase activity was remarkably decreased in cells co-transfected with circ_0007534-Wt and miR-214-3p mimics (*p* < 0.01), but was not affected in cells co-transfected with circ_0007534-Mut and miR-214-3p mimics (Figure 4C). RNA pull-down assay showed that Bio-miR-214-3p led to a higher circ_0007534 level than the treatment of Bio-NC or Bio-miR-214-3p-mut in Y-79 cells (*p* < 0.01, Figure 4D). Furthermore, cells transfected with si-circ_0007534 expressed higher miR-214-3p levels than those transfected with si-NC (*p* < 0.01, Figure 4E), indicating knockdown of circ_0007534 could increase the expression of miR-214-3p in Y-79 cells. All these results demonstrated that circ_0007534 could function as a sponge of miR-214-3p in RB. Then, the expression of miR-214-3p in RB tissues and cells was detected by qRT-PCR. The miR-214-3p levels in RB tissues were much lower than those in normal tissues (*p* < 0.001, Figure 4F). The Y-79 cells also expressed lower miR-214-3p levels than ARPE-19 cells (*p* < 0.01, Figure 4G). Finally, Y-79 cells were treated with different concentrations of osthole for 48 h, and the expression of miR-214-3p was detected by qRT-PCR. Our results showed that osthole treatment increased the expression of miR-214-3p (Figure 4H).

### The regulation of hsa_circ_0007534/miR-214-3p axis could affect osthole’s role on cell viability, proliferation, colony formation and apoptosis

**Figure 5.**
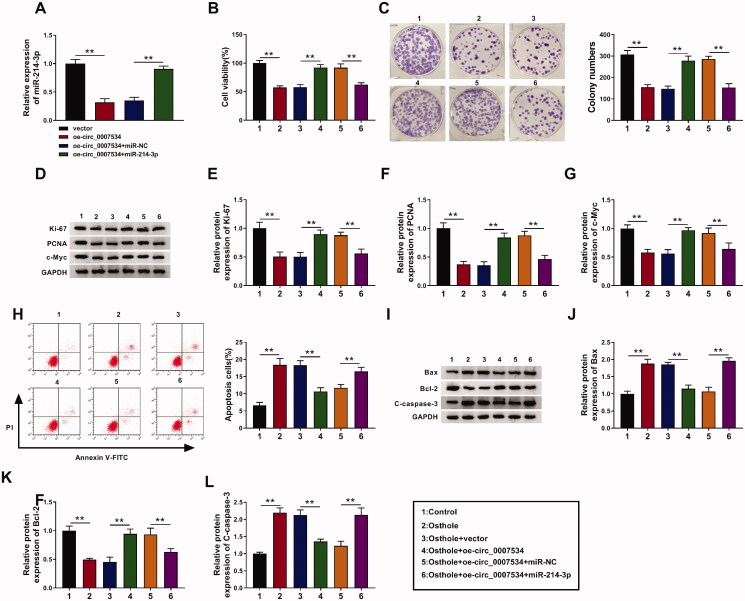
The regulation of the hsa_circ_0007534/miR-214-3p axis could affect osthole’s role on cell viability, proliferation, colony formation and apoptosis. (A) oe-circ_0007534, oe-circ_0007534 + miR-NC, oe-circ_0007534 + miR-214-3p and vector were transfected into Y-79 cells, the expression of miR-214-3p was detected by qRT-PCR. (B) The transfected and normal Y-79 cells were treated with or without osthole and then subjected to the cell viability CCK8 assay. (C) The colony-forming ability of Y-79 cells after transfection and osthole treatment was measured by clonogenic assays. (D–G) The transfected and normal Y-79 cells were treated with or without osthole, and the expression of proliferation markers was determined by western blot analysis. (H) The apoptosis rate of Y-79 cells after transfection and osthole treatment was measured by tumour cell apoptosis assay. (I–L) The transfected and normal Y-79 cells were treated with or without osthole, and the expression of apoptosis proteins was determined by western blot analysis. *p < 0.05, **p < 0.01.

Firstly, oe-circ_0007534, oe-circ_0007534 + miR-NC, oe-circ_0007534 + miR-214-3p and vector were transfected into Y-79 cells, the expression of miR-214-3p was detected by qRT-PCR. As expected, oe-circ_0007534 decreased the expression of miR-214-3p in Y-79 cells (*p* < 0.01, Figure 5A). Then, Y-79 cells were divided into six groups: control, osthole, osthole + vector, osthole + oe-circ_0007534, osthole + oe-circ_0007534 + miR-NC, and osthole + oe-circ_0007534 + miR-214-3p. Oe-circ_0007534 counteracted the inhibition effect of osthole on cell viability and colony numbers of Y-79 cells (*p* < 0.01, Figure 5B,C), as well as expressions of Ki67, PCNA and c-Myc (*p* < 0.01, Figure 5D-G). However, miR-214-3p reversed the effect of oe-circ_0007534 on cell viability, colony numbers and proliferation-related proteins levels (*p* < 0.01, Figure 5B–G). For cell apoptosis, the apoptosis rate in osthole + oe-circ_0007534 group was much lower than that in osthole + vector group (*p* < 0.01, Figure 5H). Furthermore, the levels of Bax and cleaved caspase 3 were decreased, while the level of Bcl-2 was increased in osthole + oe-circ_0007534 group as compared to the osthole + vector group. miR-214-3p also had an opposite effect against oe-circ_0007534 on apoptosis. These results indicated that regulation of the hsa_circ_0007534/miR-214-3p axis could affect the osthole’s role on cell viability, proliferation, colony formation and apoptosis.

### Osthole inhibited tumour growth *in vivo*

**Figure 6.**
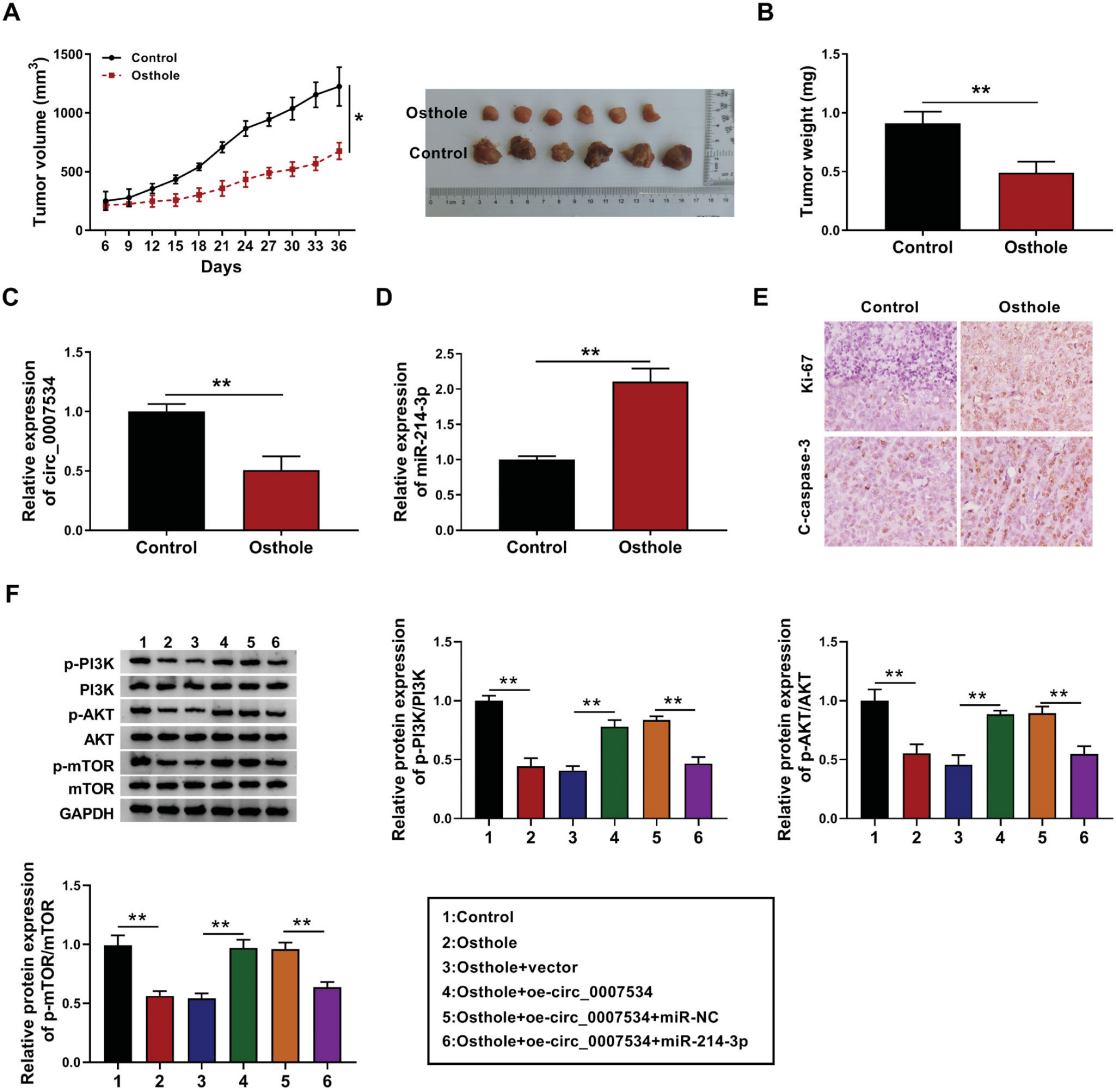
Osthole inhibited tumour growth *in vivo*. (A) Tumour volume was calculated in osthole group and control group every 3 days. (B) Tumour weight was analysed after 36 d of injection. (C) Relative expression of has_circ_0007534 was detected in osthole group and control group. (D) Relative expression of miR-214-3p was detected in osthole group and control group. (E) The expression of Ki-67 and cleaved caspase 3 in osthole group and control group was detected by immunohistochemistry. (F) Y-79 cells were transfected with oe-circ_0007534, vector, oe-circ_0007534 + miR-NC and oe-circ_0007534 + miR-214-3p, respectively. And tumour bearing mice were established using these cells and treated with osthol. After 4 weeks, mice were sacrificed, and the expression of the protein in PI3K/AKT/mTOR pathway was detected by western blot. *p < 0.05, **p < 0.01.

Based on the *in vitro* results, we further validated the effect of osthole on RB *in vivo.* Our results indicated that osthole significantly delayed the tumour growth of the mice (Figure 6A,B). Consistent with the *in vitro* results, osthole decreased the expression of hsa_circ_0007534 (*p* < 0.01, Figure 6C) and increased the level of miR-214-3p *in vivo* (*p* < 0.01, Figure 6D). In addition, IHC was used to assess the expression of Ki67 and cleaved caspase 3 in tumours. Compared with the control group, Ki67 was significantly decreased, whereas cleaved caspase 3 was increased in osthole treatment group (Figure 6E), indicating osthole could inhibit RB cell proliferation and induce RB cell apoptosis *in vivo*.

Then, 30 mice were recruited to determine the role of osthole on PI3K/AKT/mTOR pathway *in vivo*. Y-79 cells were transfected with oe-circ_0007534, vector, oe-circ_0007534 + miR-NC and oe-circ_0007534 + miR-214-3p, respectively. And tumour bearing mice were established using these cells and treated with osthole. After 4 weeks, mice were sacrificed, and the expression of the protein in PI3K/AKT/mTOR pathway was detected by western blot. Compared to the control, osthole decreased the ratios of p-PI3K/PI3K, p-AKT/AKT and p-mTOR/mTOR (*p* < 0.01, Figure 6F). However, hsa_circ_0007534 overexpression reversed the effect of osthole on PI3K/AKT/mTOR pathway. And miR-214-3p could counteract hsa_circ_0007534’s effect on this pathway (Figure 6F). These results confirmed that osthole inhibited the PI3K/AKT/mTOR pathway via hsa_circ_0007534/miR-214-3p axis.

## Discussion

In the present study, we found that osthole inhibited cell viability, proliferation, colony formation and induced apoptosis in RB cells through PI3K/AKT/mTOR pathway. Hsa_circ_0007534 was relatively highly expressed in RB tissues and cells, which could sponge miR-214-3p to regulate RB cell proliferation and apoptosis. Osthole treatment reduced the expression of hsa_circ_0007534, while increasing the expression of miR-214-3p. Thus, osthole might exhibit its antitumor activity for RB by inhibiting PI3K/AKT/mTOR pathway via regulating the hsa_circ_0007534/miR-214-3p axis.

Osthole has been reported to induce apoptosis and have anti-proliferative activity in a variety of human cancer cells, including breast (Park et al. 2019), gastric (Xu et al. 2018), ovarian (Bae et al. 2021), and cervical cancers (Che et al. 2018). The results of the present study extend the antitumor activity of osthole to RB. Osthole treatment inhibited the growth of Y-79 cells in this study. And the expression of proliferation markers (Ki67, PCNA, and c-Myc) (Kong et al. 2020) could also be reduced by osthole. It is well known that the induction of tumour cell apoptosis is a crucial component of antitumor therapeutic agents (Che et al. 2018). Caspase-3 is a key protease in mitochondria-dependent and -independent apoptosis pathways, executing the final phase of apoptosis (Ma et al. 2020). Bcl-2, in contrast to Bax, is an anti-apoptotic protein in the mitochondrial apoptosis pathway (Che et al. 2018). Our results demonstrated that the osthole-induced RB cell apoptosis was mediated by modulating the expression of the activity of Bcl-2, Bax and cleaved caspase-3.

The PI3K/AKT/mTOR signalling pathway plays a crucial role in the growth of various kinds of tumours including RB (Chai et al. 2017). Following PI3K activation, AKT is phosphorylated, and p-AKT subsequently activates the mTOR, regulating cell growth, proliferation, motility, and survival (Zhang and Fan 2020). The inhibiting effect of osthole on PI3K/AKT pathway has been observed in previous studies (Xu et al. 2018; Zhu et al. 2018). The data from the present study strongly supported these findings. 740Y-P is a frequently used PI3K agonist (Qu et al. 2019). In this study, we found that 740Y-P could reverse the effects of osthole on proliferation markers and apoptosis proteins, further indicating that osthole affected the growth of RB cells via PI3K/AKT/mTOR pathway.

Hsa_circ_0007534 was a recently discovered cancer-associated circRNA. Elevated expression of hsa_circ_0007534 has been found in several tumour types including CRC (Zhang et al. 2018) and non-small cell lung cancer (Qi et al. 2018). Hsa_circ_0007534 could increase cancer cell proliferation, migratory, invasion, and inhibit cell apoptosis (Hao et al. 2019). Downregulation or knockdown of hsa_circ_0007534 has been reported to restrict the proliferation and invasion of cancer cells significantly (Song and Xiao 2018; Rong et al. 2019; Ding et al. 2020). The link between hsa_circ_0007534 expression and RB has not been determined previously. Herein, we showed that hsa_circ_0007534 was significantly upregulated in RB tissues and RB cells, indicating that hsa_circ_0007534 is a potential cancer-related gene in RB. Our RNase R and actinomycin D assays indicated that hsa_circ_0007534 was a circular and stable transcript. Previous studies suggest that circRNAs may function as miRNA sponges, translate to proteins, or interact with RNA-binding protein to regulate their downstream targets (Dori and Bicciato 2019; Sun et al. 2019). Our cell fraction assay revealed that hsa_circ_0007534 was mainly located in the cytoplasm, indicating that it may act as miRNA sponges. miRNAs, with 19–25 nucleotides in length, were identified as a family of small non-coding RNAs that modulated the level of the target gene by inhibiting translation or inducing degradation of mRNA in human cancers (Ding et al. 2020). Previous studies demonstrated that hsa_circ_0007534 exerted its function by repressing the expression of several miRNAs including miR-613, miR-498 and miR-593 (Song and Xiao 2018; Rong et al. 2019; Ding et al. 2020). In the present study, miR-214-3p was validated as a direct target of HNF1A-AS1 based on the following results: firstly, bioinformatics tools showed a putative targeting site of miR-214-3p in hsa_circ_0007534 transcript; secondly, the luciferase activity and RNA pull-down assays revealed the direct binding of miR-214-3p to hsa_circ_0007534; finally, the knockdown of circ_0007534 increased the expression of miR-214-3p in Y-79 cells directly.

In the present study, we also found a link between osthole treatment and the hsa_circ_0007534/miR-214-3p axis. As expected, osthole treatment reduced the expression of hsa_circ_0007534, while increasing the expression of miR-214-3p, indicating osthole had a regulating effect on hsa_circ_0007534/miR-214-3p axis. Y-79 cells overexpressed hsa_circ_0007534 were resistant to osthole’s effect on cell viability, proliferation, colony formation and apoptosis. However, miR-214-3p reversed this condition.

The *in vivo* antitumor effect of osthole on RB was also explored in this study. Our data showed that osthole significantly inhibited tumour growth in RB xenograft mouse models. Furthermore, consistent with our *in vitro* results, osthole also decreased the expression of Ki67 and increased the expression of cleaved caspase 3 *in vivo*. Previous studies demonstrated that circRNAs could affect the phosphorylation of PI3K/Akt/mTOR signalling pathway components. Wang et al. (2020) found that circEPSTI1 sponged miR-942-5p to accelerate EMT in oral squamous cell carcinoma through phosphorylation of PI3K/Akt/mTOR signalling pathway. Peng et al. (2020) demonstrated that hsa_circ_0010882 played an important role in proliferation, migration, and invasive genotypes of gastric cancer cell lines via regulation of the PI3K/Akt/mTOR signalling pathway. In the present study, we found osthole could inhibit the phosphorylation of PI3K/Akt/mTOR signalling pathway components *in vivo*. However, hsa_circ_0007534 overexpression could reverse the effect of osthole on PI3K/AKT/mTOR pathway. And miR-214-3p could counteract hsa_circ_0007534’s effect on this pathway. The *in vivo* results confirmed that osthole inhibited the PI3K/AKT/mTOR pathway via regulating the hsa_circ_0007534/miR-214-3p axis.

## Conclusions

Our findings suggested that osthole exhibited an antitumor effect in RB through inhibiting PI3K/AKT/mTOR pathway via regulating the hsa_circ_0007534/miR-214-3p axis. Consequently, we propose that osthole may serve as a potential therapeutic agent in RB treatment.

## Data Availability

The analysed data sets generated during the study are available from the corresponding author on reasonable request.
